# The mechanism of balloon Eustachian tuboplasty: a biomechanical study

**DOI:** 10.1007/s11517-020-02121-z

**Published:** 2020-01-17

**Authors:** Matthew E. Smith, Anna E. Weir, Daisy C.C. Prior, Wei Cope, James R. Tysome, Michael Sutcliffe

**Affiliations:** 1 Cambridge Ear Institute, Cambridge Biomedical Campus, Cambridge, CB2 0QQ UK; 2 Engineering Department, University of Cambridge, Trumpington Street, Cambridge, CB2 1PZ UK; 3 Department of Pathology, Addenbrooke’s Hospital, Cambridge Biomedical Campus, Cambridge, CB2 0QQ UK

**Keywords:** Eustachian tube, Dilation, Balloon, Pressure, Deformation, Mechanics, Histology

## Abstract

Obstructive Eustachian tube dysfunction (OETD) is a common condition resulting from inadequate opening of the Eustachian tube (ET). A new surgical treatment involves high-pressure inflation of a balloon within the ET, with the aim of dilating the soft tissue structure. However, the mechanical effects of this intervention have not been established, nor the impact of changing device size or other technical parameters. A novel experimental technique allowed quantification of plastic and elastic tissue deformation in model materials and then human cadaver ETs during balloon dilation, based on the measured balloon inflation pressure-volume relationship. Plastic tissue deformation was found to be greater using larger balloons and deeper device insertion, but increasing the inflation pressure had a more limited effect, with most deformation occurring well below the clinically used pressures. Histological assessment of ET tissue suggested that mucosal tearing and cartilage cracking were in part responsible for the mechanical changes. Balloon dilation of the ET has huge potential if found to be clinically effective, but currently there is a need to understand and develop the technique further. The novel methods employed in this study will be valuable in future laboratory and in vivo studies of ET balloon dilation. Pressures are reported in Bar as this unit is used for medical balloon dilation procedures in clinical practice. 1 Bar = 100,000 Pa.

**Electronic supplementary material:**

The online version of this article (10.1007/s11517-020-02121-z) contains supplementary material, which is available to authorized users.

**Graphical abstract caption:**
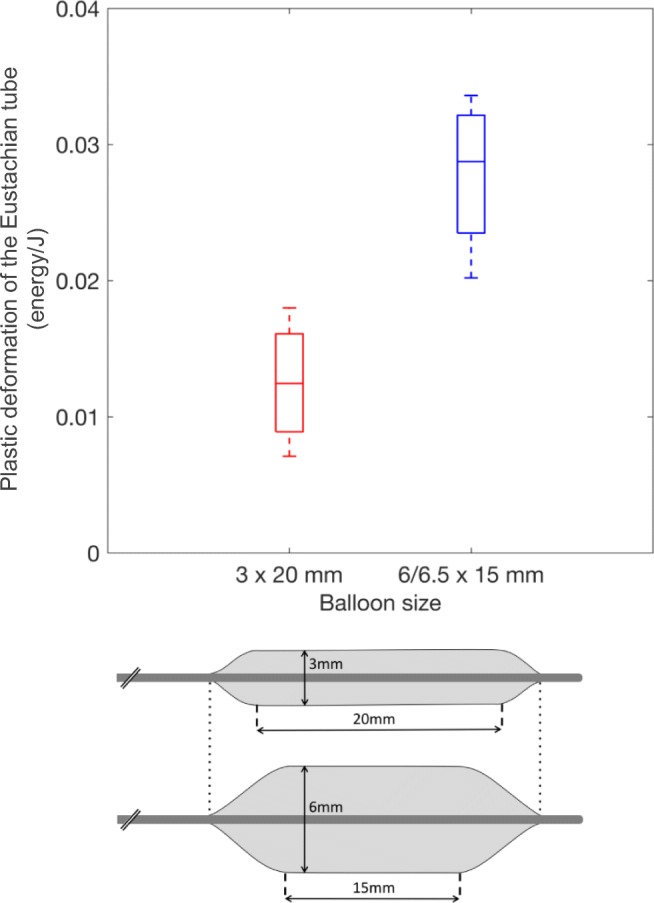
Dilation of the Eustachian tube for obstructive dysfunction is performed clinically with 3- and 6-mm-diameter balloons of approximately the same overall length. Our data suggest that dilation with a 6-mm balloon causes greater deformation of the soft tissue structure than dilation with a 3-mm balloon. This difference has yet to be demonstrated clinically. Plastic deformation was measured in terms of energy (J) dissipated during balloon inflation.

## Introduction

Obstructive Eustachian tube dysfunction (OETD) is a common condition resulting from inadequate opening of the Eustachian tube (ET), a structure that is crucial in the ventilation pathway of the gas-filled middle ear. Symptoms of OETD affect approximately 0.9% of the UK adult population, but associated middle ear disorders are seen in significantly higher numbers [1]. Balloon Eustachian tuboplasty (BET) is a minimally invasive surgical procedure that is increasingly being adopted as a treatment for OETD. Early clinical results appear to indicate that the procedure is an effective means to reduce symptoms and normalise middle ear pressure [2], in a condition where other medical and surgical interventions have been found ineffective [3].

The principle of dilatation to open an obstructed tube or orifice has been successfully applied for many years in blood vessels and other structures [4]. However, the ET is a dynamic tubular structure that must remain closed at rest and open during paratubal muscle contraction. The part of the ET treated by BET is formed largely from a glandular soft tissue lining (mucosa and submucosa) and a cartilage skeleton, shaped like an inverted-J, that arches over the tube along its length.

Little work has been done to establish what the mechanism for the clinical improvements seen with BET may be. The limited number of non-clinical studies of BET has suggested that crushing, cracking and tearing of the mucosa and cartilage may occur [5–8], although reports are not consistent, and it has not been investigated how these changes may affect ET opening. Although competitor BET devices are now available, currently the clinical evidence relates to two balloon devices (and their precursors) that vary in both length and cross-sectional area, the latter by a factor of 4. A lack of standardisation in the surgical technique also exists, with different balloon inflation pressures and single or multiple inflations being adopted. Study heterogeneity has prevented analysis of the impact of variation in device or technique on clinical outcomes. As further clinical data emerges, an understanding of the mechanical and traumatic effects of the device size, inflation pressure and number of inflations may allow refinement of the procedure. A greater understanding of the mechanism of BET may also permit direction of the technique to subgroups of patients who would benefit most, as OETD varies in aetiology, presentation and severity [9].

If BET is to improve ET function, this must be due to mechanical action of the BET balloon, either directly improving ET opening, or causing trauma that leads to beneficial tissue or architectural changes. This study aimed to establish if BET could be linked to changes within the structure or mechanical properties of the ET, and how differences in the BET device or technique may affect the changes occurring.

## Materials and methods

The use of human cadaveric material in this study was approved by a sub-committee of the West of Scotland Research Ethics Committee.

### Pressure-volume measurements

**Fig. 1:**
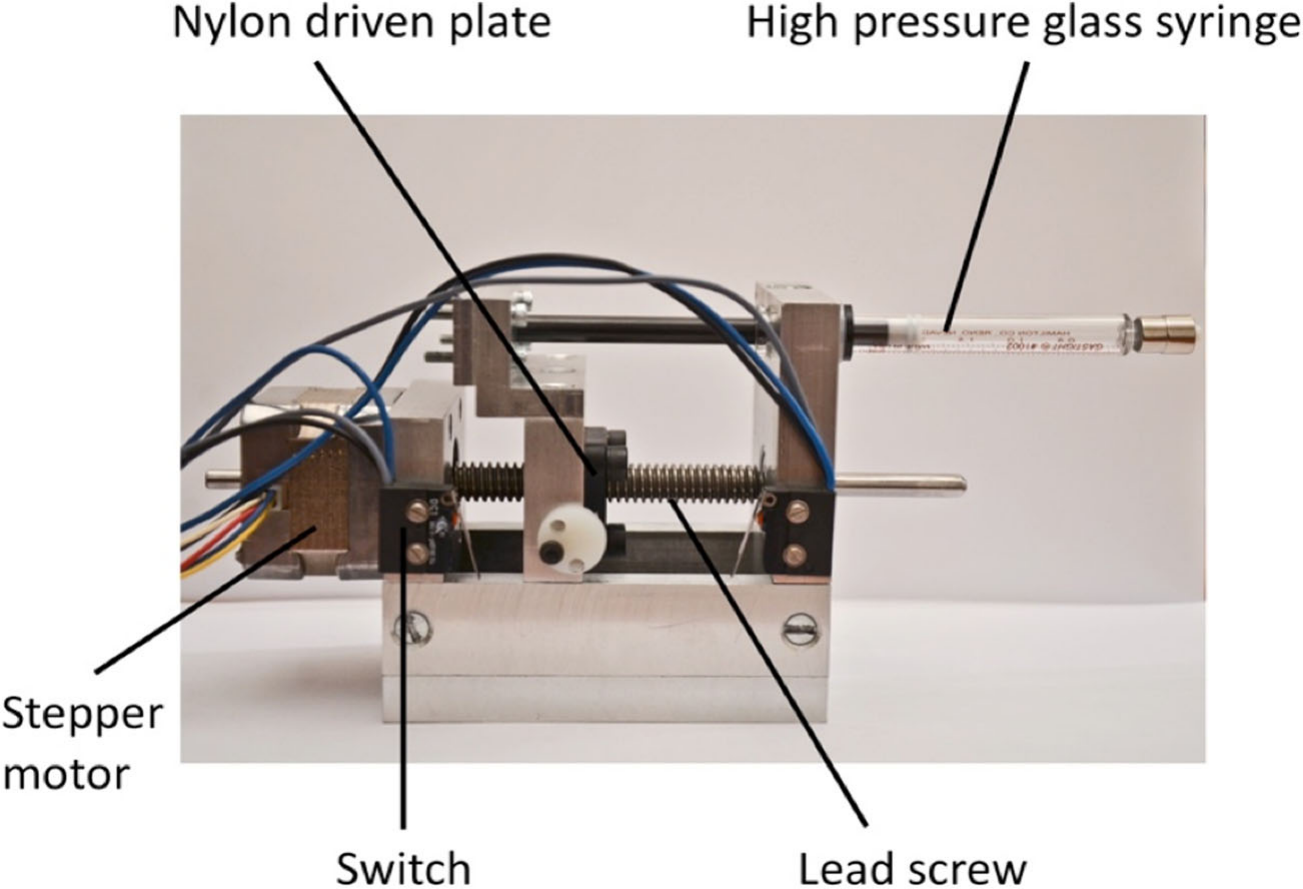
Precision high-pressure syringe driver

To allow controlled balloon inflation, a bespoke high-pressure precision syringe driver was constructed to be capable of generating pressures up to 10 bar, driven by a stepper motor (Fig. 1).

A glass syringe (2.5 mL 200 psi, Hamilton, Cinnaminson, NJ) was connected via a 3-way luer-lock connector to a pressure sensor (250 psi, accuracy 0.2%, Honeywell, Morristown, NJ) and the balloon catheter under investigation. Sensor output was recorded on a laptop via an amplifier and data acquisition device (NI USB-6009, National Instruments, Austin, TX). The pressure sensor was calibrated prior to use, and accuracy confirmed at intervals. The volume delivered by each motor step was calculated using component specification values, and confirmed experimentally. The catheter, syringe and all connectors were filled with water, with care taken to purge any air bubbles. Motor control and pressure data recording were performed in LabVIEW 2015 (National Instruments).

### Balloon devices

Three devices were assessed: the 3.0 × 20 mm TubaVent BET catheter (Spiggle & Theis Medizintechnik GmbH, Overath, Germany), the 6.0 × 15 mm EverCross angioplasty catheter (Covidien/Medtronic Ltd., Dublin, Ireland), and the 6.5 × 15 mm Sterling Monorail, angioplasty catheter (Boston Scientific, Marlborough, MA), the latter two acting as a similarly sized and structurally comparable alternative to the AERA BET device (Acclarent Inc.) which was unavailable at the time of the study. Prior to first use, each new catheter was inflated to working pressure once with no data collected, in order to unfold the packaged balloon. Preliminary studies confirmed that after the device’s first use, subsequent inflations were indistinguishable in terms of the pressure-volume relationship during inflation. Balloon diameter was measured three times using Vernier callipers placed across both the proximal and distal end of the parallel segment of the balloon, and a mean diameter value calculated for each of four inflation pressures (2.5, 5.0, 7.5 and 10.0 bar). Balloon overall length including the tapered sections (measured at 10 bar inflation pressure) was also measured with callipers three times, with a mean calculated.

### Cadaver and model material specimens

BET was performed in human cadavers, and in simplified ET structures made from model materials. Adult fresh-frozen cadaveric heads were thawed overnight, after which they have tissue properties close to those found in vivo. To explore ET deformation during BET, model materials with purely plastic or elastic properties were selected. Silicone tubing of inner diameter (ID) 2 mm and outer diameter (OD) 4 mm was used as an elastic model of the ET, with detergent (Fairy, Procter and Gamble) used to lubricate the balloon within the tube. White plasticine (Newplast, Newclay Products) formed into tubes of ID 2 mm and OD 5 mm using a custom mould was used as a plastic model of the ET.

### Pressure-volume analysis of BET

Three protocols were used in different specimens. Each Eustachian tube was only used once for a single protocol.

(1) The balloon catheter was inflated from 0 to 10 bar before immediate deflation to 0 bar, forming an inflation-deflation cycle. The cycle was initially performed outside the cadaver specimen, with the balloon unconstrained. The BET catheter was then inserted into the cadaver or model material ET, in the cadaver using a 45° angled introducer guided via a 30° endoscope. The catheter was slowly advanced into the tube, stopping to reposition if any resistance was felt. Once the catheter was fully inserted, three 10-bar inflation-deflation cycles were performed, with approximately 1 min between each, without moving the catheter between cycles. This protocol was used for both 3- and 6-mm balloons in both the cadavers and the two models. For this protocol, most cadavers received BET on one side/ear, with the other side untouched. In most cases, each cadaver had a 3.0-mm diameter balloon inflated in one ET, and a 6.0- or 6.5-mm balloon inflated on the other side, generating paired data.

(2) As in protocol 1, a 10-bar inflation-deflation cycle was performed with the balloon unconstrained, and the catheter then inserted into the ET. The balloon was first inflated to 2.5 bar, before immediate deflation. The 2.5-bar inflation-deflation cycle was repeated twice further. Without moving the balloon, further inflation-deflation cycles to pressures of 5, 7.5 and 10 bar were used, with cycles at each pressure repeated three times. This protocol was only used in the human cadavers. Each cadaver had a 3.0-mm-diameter balloon inflated in one ear ET, and a 6.5-mm balloon inflated on the other side.

(3) Following an unconstrained 10-bar inflation-deflation cycle, further cycles were performed at different ET insertion depths. Using paint marks on the balloons, the central, parallel sections of the 3.0 × 20 mm balloons were inserted to depths of 10, 15 and 20 mm from the nasopharyngeal ostium, while the 6.0-mm balloons were inserted to 10 and 15 mm. During initial inflations, the balloons therefore protruded, unconstrained, within the nasopharynx. As with protocol 2, this protocol was only used in human cadavers, and in all cases 3.0- and 6.0-mm balloons were used on opposite sides.

### Pressure-volume data analysis

Data recorded for all inflation-deflation cycles were analysed in Matlab (MathWorks, Inc.). For each cycle, inflation pressure was plotted against injected volume, tending to form a loop due to the hysteresis of the system.

The area under the inflation part of the pressure-volume loop represents the energy required to inflate the balloon against resistance. If the balloon is unconstrained, this energy is the work done on the balloon’s structure alone, whereas during BET, it is the work done on the balloon and the ET. The area under the deflation part of the loop is the energy returned by the tissues and balloon as they return to their original shape. The area within the hysteresis loop therefore represents the energy dissipated into the balloon and ET. To calculate the energy dissipated into the ET during plastic deformation, the area within the 2nd cycle pressure-volume loop, representing elastic deformation alone, was subtracted from the area within the 1st cycle loop (Supplementary Figure A).

The energy transferred to the ET soft tissues through plastic deformation was considered to approximate the degree of plastic deformation occurring during BET, and was compared between the two balloon sizes.

### ET staining, harvesting and histology

Separate to the pressure-volume studies, some cadaver ETs were histologically examined after BET. ETs were flushed bilaterally with saline, before 2 ml of Alcian blue stain (aqueous solution) was injected into each ET using a soft-tipped syringe, and then confirmed to enter the middle ear. Next a 3.0 × 20 mm or 6.0 × 15 mm balloon catheter was inserted into the left or right ET, and a single 10-bar inflation-deflation cycle was performed. The contralateral ET was not treated, acting as a control. ETs were then thoroughly irrigated with saline to remove residual stain.

ETs were harvested via a trans-oral approach, having removed the soft palette. Blunt and sharp dissections were used to free the ET from the skull base and surrounding soft tissue structures, taking care not to crush the specimen. The cartilaginous ET was transected free as close to the bony portion as was technically possible. Specimens were fixed in 10% neutral buffered formalin, and cut transversely at 4-mm intervals for standard histological processing. Sections were prepared with haematoxylin and eosin stains.

## Results

Measured balloon diameters were close to the manufacturer stated values. With increasing inflation pressure between 2.5 and 10 bar, the 3.0 mm, 6.0 mm and 6.5 mm the unconstrained balloons increased in diameter by 0.39, 0.58 and 0.92 mm, respectively. The diameter at each pressure can be seen in Supplementary Figure B. Despite the stated length of the parallel segment of the balloons differing by 5 mm, the overall balloon length, including tapered sections, was remarkably similar for the test devices: 26.50 mm, 26.42 mm and 24.74 mm for the 3.0 mm, 6.0 mm and 6.5 mm balloons, respectively.

Protocol 1 was completed using the 3.0-mm balloon in four plasticine tube models and four silicone tube models. The ET was dilated according to protocol 1 on both sides in seven cadaver heads, using the 3.0-mm balloon on one side, and the 6.0- or 6.5-mm balloon on the contralateral side. Data from one 6.5-mm-diameter balloon was interrupted and unusable.

**Fig. 2:**
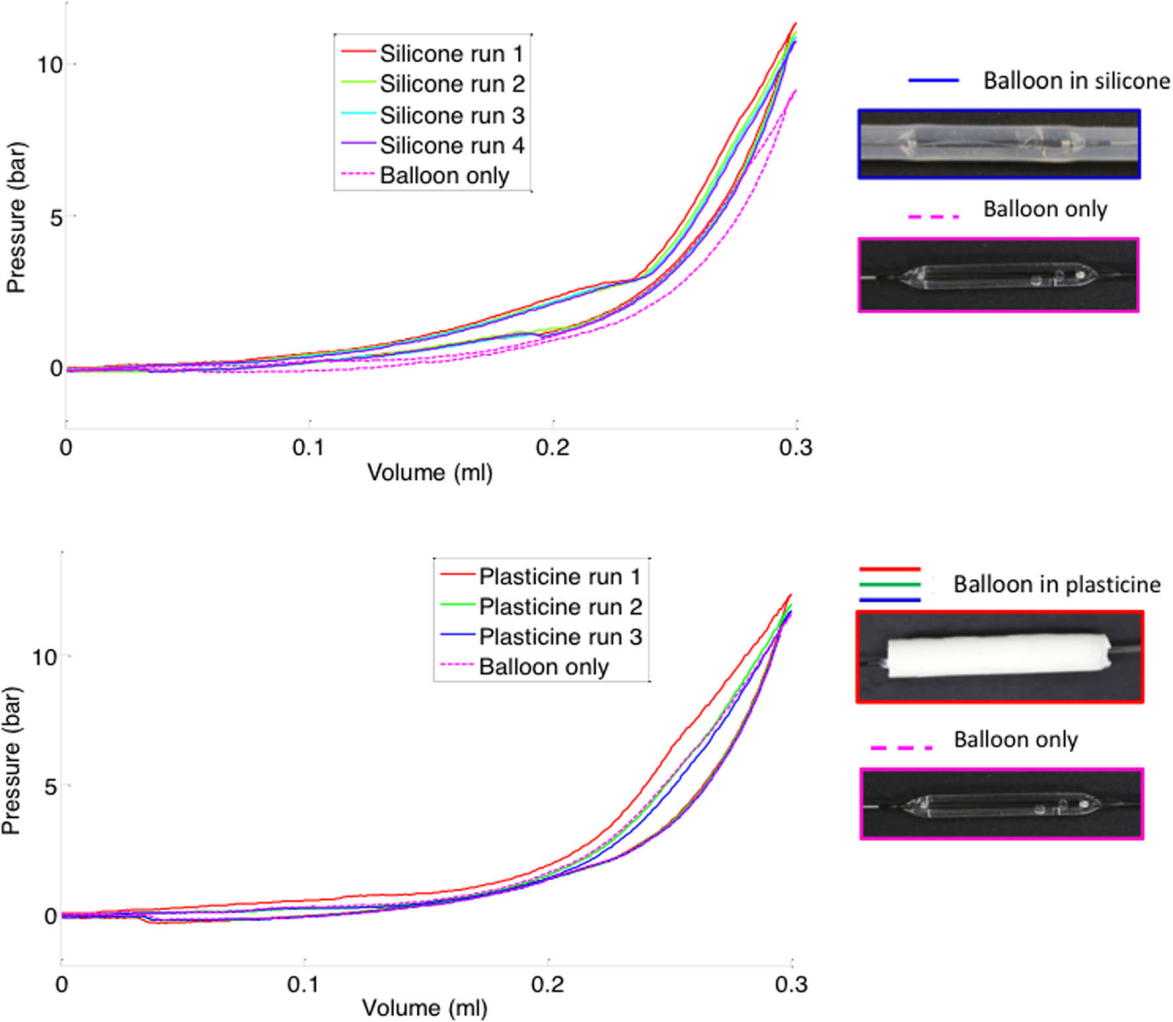
Example pressure-volume loops obtained from BET at 10 bar in silicone (top) and plasticine (bottom) model materials

For both the silicone and plasticine ET models, on the first inflation the pressure within the balloon at any inflation volume was greater with the balloon within the model ET lumen (constrained), as opposed to when it was performed outside the model (unconstrained) (Fig. 2). For the plasticine model, the pressure was only higher than the unconstrained baseline during the first inflation, during which plastic, non-reversible deformation took place. In subsequent cycles, pressure-volume loops all overlaid the unconstrained loop, as the permanently deformed plasticine model no longer exerted any constraining force. For the silicone model, the pressure was higher than the unconstrained baseline for all inflation-deflation cycles, with no difference between the first and subsequent cycle loops, as all deformation occurring both in the balloon and surrounding silicone tube was reversible on balloon deflation.

**Fig. 3:**
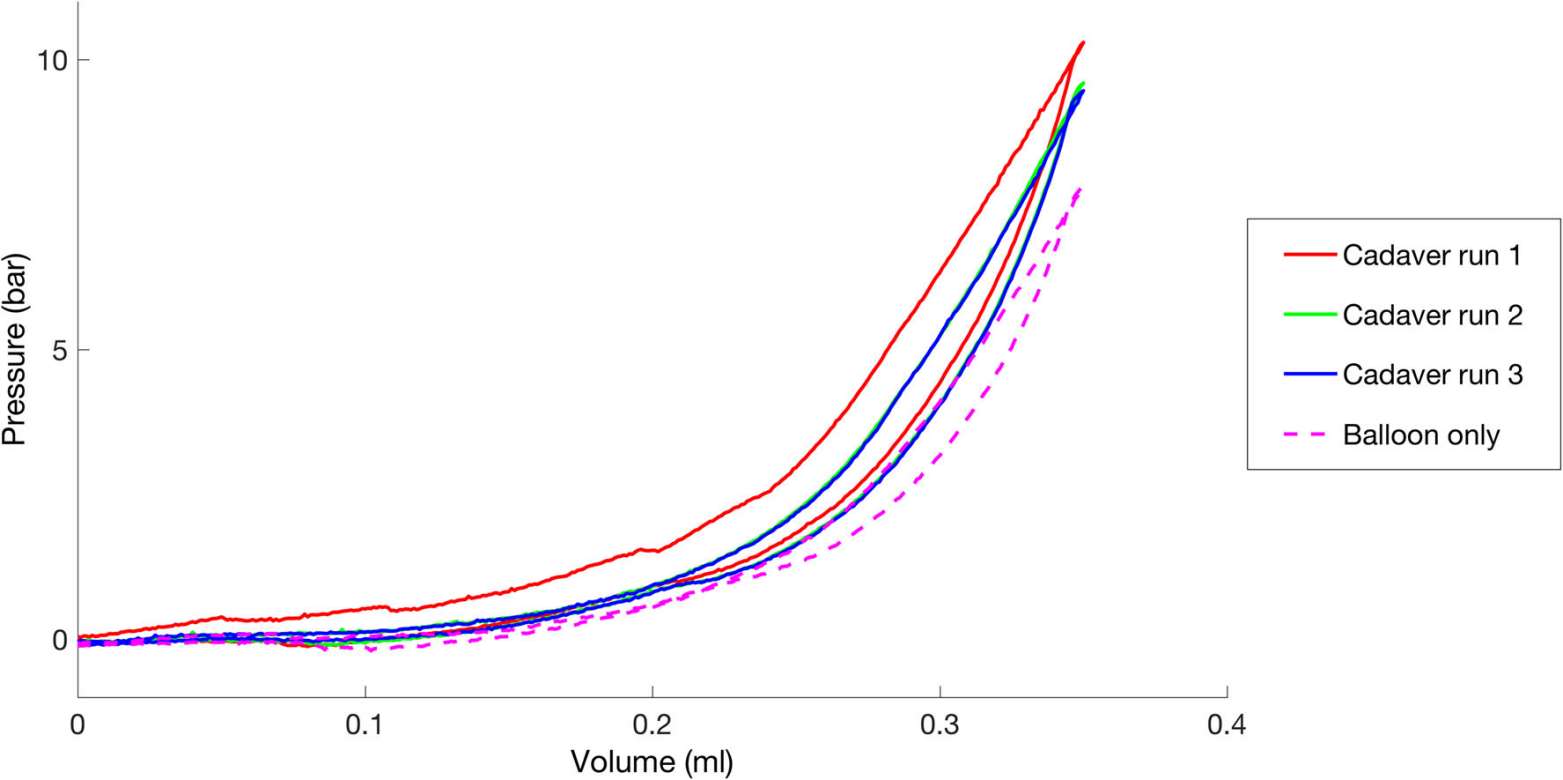
Pressure-volume loops for an example 3 mm balloon in a cadaver ET. Three distinct types of pressure-volume loop can be seen; the first cycle, subsequent cycles (2&3) and a balloon-only unconstrained inflation

Pressure-volume loops from cadaveric samples dilated using protocol 1 all displayed certain common features (example, Fig. 3). In all cases, and for both 3.0-mm and 6.0/6.5-mm balloons, the pressure during inflation at every volume was highest during the first inflation-deflation cycle, suggesting that like the plasticine, plastic deformation occurred during this cycle. In all cases, the second and third cycles produced similar pressures that were lower than those during the first cycle, but higher than those during the unconstrained inflation-deflation. This suggested there was elastic deformation of the ET occurring during all inflations, as seen with the silicone model.

**Fig. 4:**
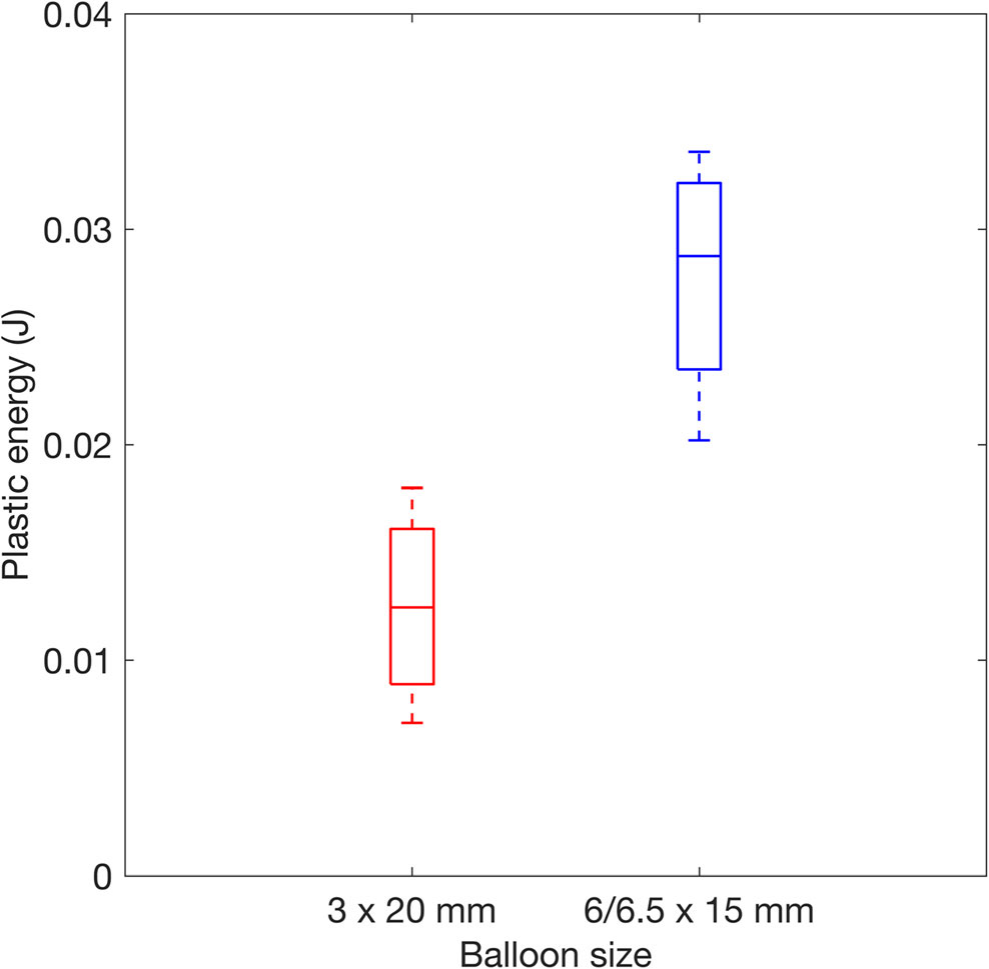
Plastic energy dissipation (work done in plastic deformation) during BET with 6 × 15 mm and 3 × 20 mm diameter balloons. *n* = 5 paired data are included (from opposite ears in the same cadaver head). On each box, the central mark indicates the median, and the bottom and top edges of the box indicate the 25th and 75th percentiles, respectively. The whiskers extend to the most extreme data points

The energy transferred to the ET tissues through plastic deformation was compared for the two different BET balloons (Fig. 4). The values found from different ears were consistent (indicated by the box plot whiskers), and despite a small sample size, the difference in mean value between the two balloon sizes was statistically different using a paired two-sided *t* test (*p* = 0.0055), suggesting that more deformation occurred with the 6.0/6.5 × 15 mm balloon.

**Fig. 5:**
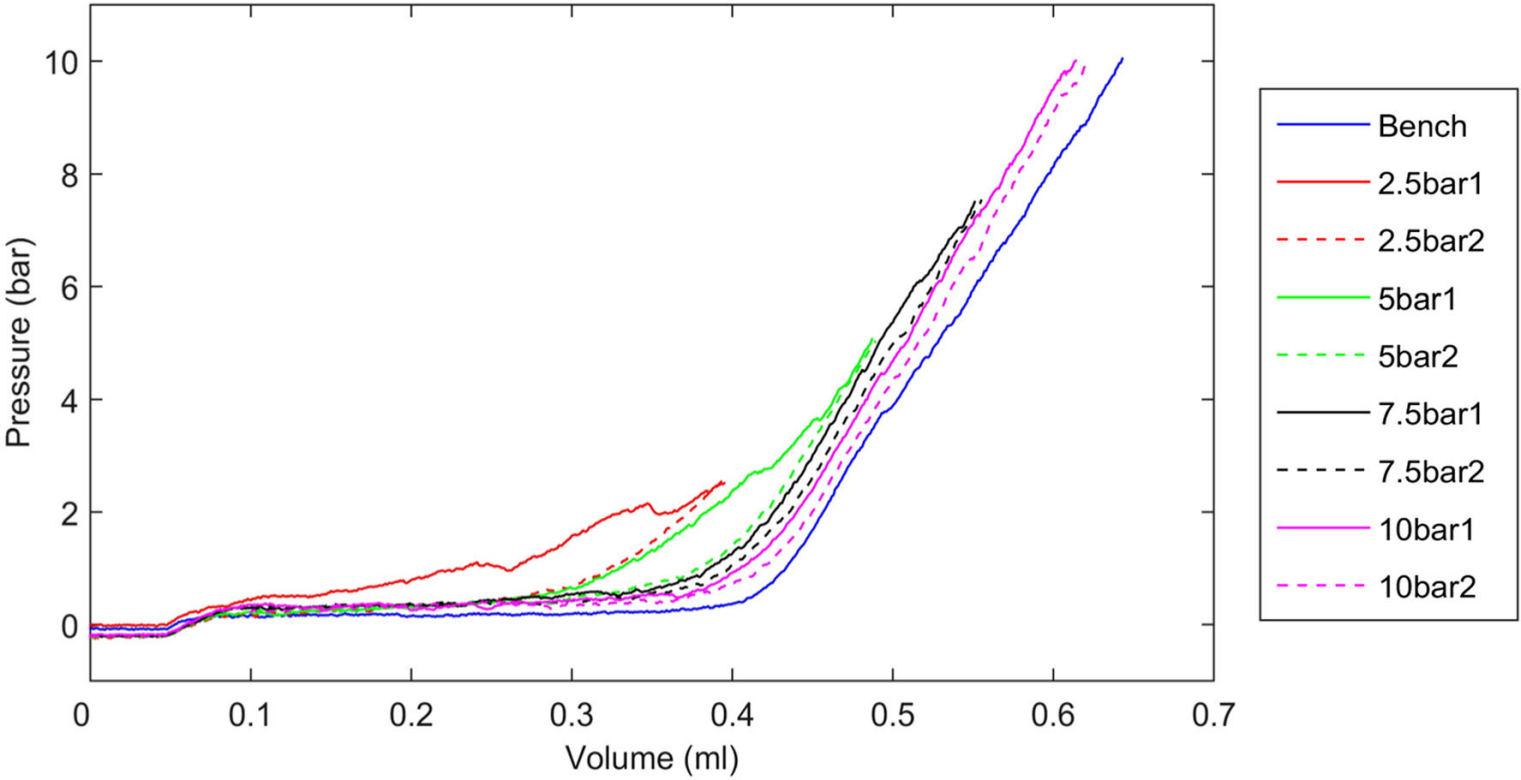
Pressure-volume loops (with deflation stage removed for clarity) for an example 6.5 × 15 mm balloon in a cadaver ET. Pressure was increased in 4 stages (protocol 2). A different colour has been used for each inflation pressure. The solid line represented the first inflation at each pressure, and the dashed line of the same colour is the second inflation to that pressure. The third inflation had a similar pressure-volume relationship to the second

Protocol 2 was used in 10 ears (5 cadavers). The difference in pressure-volume loops between the first and second cycles constrained within the ET was greatest for the 2.5 and 5 bar cycles (example Fig. 5), suggesting that the majority of the ET plastic deformation occurred at lower inflation pressures. With the 7.5 and 10 bar cycles, the difference between the first and second constrained inflations was limited, suggesting that mainly elastic deformation occurred at higher pressures.

**Fig. 6:**
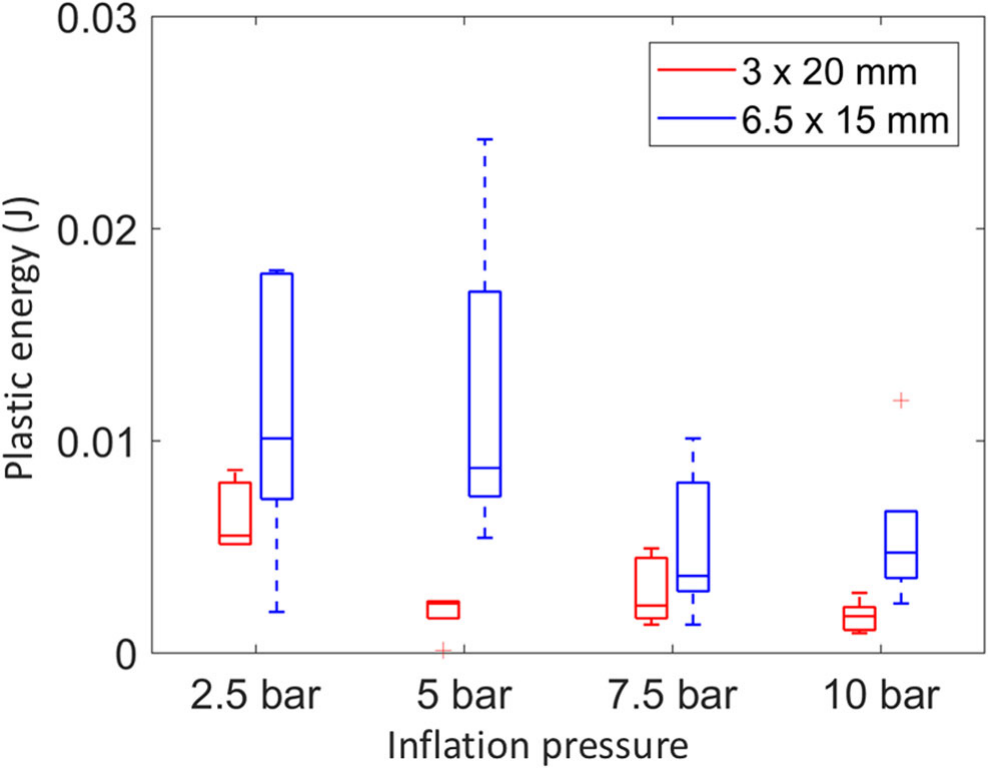
Plastic energy dissipation (work done in plastic deformation) for 3.0 × 20 mm and 6.5 × 15 mm balloons (performed in contralateral ears) in 5 heads. Box plots as per Fig. 4

The energy dissipated in plastic deformation was calculated for the four different inflation pressures (Fig. 6). Based on the principle that all plastic deformation occurs on the first inflation to a given pressure, the energy relating to different stages of inflation could be determined. For example, as inflation to 5 bar had already been performed, the energy dissipated on the 7.5-bar inflation corresponded to the deformation occurring between 5 and 7.5 bar. For both balloon sizes, there was a trend for less energy dissipation at higher inflation pressures, with the majority of the energy dissipation, and therefore plastic deformation, occurring below 7.5 bar for the 6.5-mm balloons, and below 5 bar for the 3.0-mm balloons.

Protocol 3 was used in 6 ears (3 cadavers). For the 3.0-mm balloon, there was a trend for plastic deformation to increase with depth of insertion, with little occurring in the first 10 mm of the ET closest to the nose (in all three cases the pressure-volume loops at 10-mm insertion depth did not vary from the unconstrained loop). At 15 and 20 mm insertion depth, the 3.0-mm balloon pressure-volume loops consistently suggested deformation. For the 6.0 × 15 mm device, in all three cases, plastic deformation appeared to occur at both 10- and 15-mm depths. A small sample size prevented reliable analysis of energy dissipation.

### Histology

**Fig. 7:**
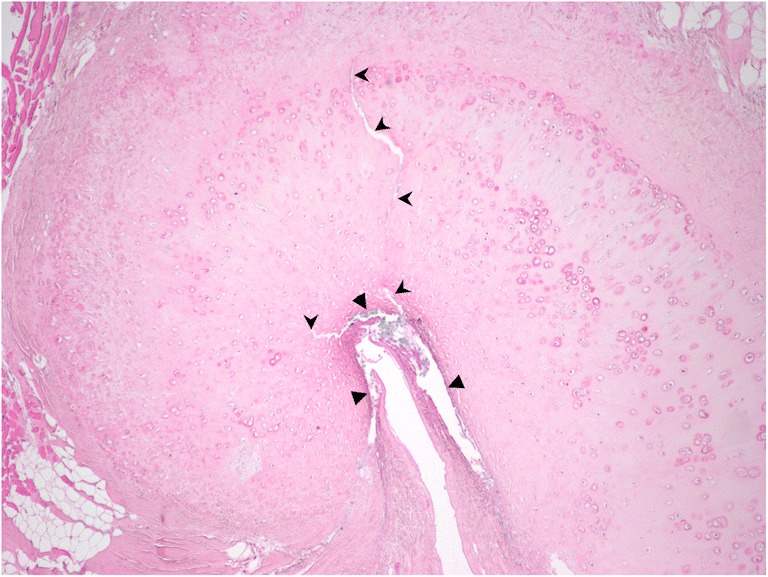
Histological section showing the apex of the curved ET cartilage in a BET-treated ear. Triangles indicate areas of mucosal tearing and shearing from the submucosa. Arrowheads indicate two cracks in the cartilage radiating from the apex of the ET lumen. Haematoxylin and eosin stain with Alcian blue stain introduced into the ET lumen before BET. ×4 magnification

Staining and ET harvest was performed in 12 ears (6 cadavers) with each head undergoing BET on one side, with either a 3.0- or 6.0-mm balloon. One ET could not be successfully harvested and histological sections from another ET were not useable due to calcification. No cartilage cracks or mucosal tears were present in any of the control ETs, whereas cracks containing Alcian blue stain were present in 3/5 BET-treated samples (example Fig. 7). All cracks occurred at the apex of the J cartilage, in what is considered the hinge portion. 4/5 BET samples contained mucosal tears, two with stain trapped below and within the mucosa.

## Discussion

BET is a relatively new intervention, at an early stage of development. Clinical evidence from well-designed trials using clinically relevant outcome measures is very limited, and the first priority with the technique must be to establish a beneficial clinical effect in ears with OETD. A second priority is to understand the mechanism of BET, and to optimise the technique and devices to maximise any clinical effect.

Despite increasingly widespread clinical adoption of BET as a treatment for OETD, the mechanism through which it may improve ET function remains unknown. Previous cadaveric studies have suggested that structural changes occur in the ET during BET, resulting in a greater than three-fold increase in ET luminal volume [6], and reduced passive ET opening pressure following BET [10]. However, the mechanical effect of BET on the ET has not been characterised before, and neither has the impact of varying balloon size or altering aspects of the technique.

### What happens to the ET during BET?

Comparing the cadaver data to the model material data suggests that the ET undergoes both plastic and elastic deformation during BET, and it was found to consistently occur in all ears with both the 3.0- and 6.0/6.5-mm-diameter balloons.

The finding of plastic deformation of the ET during BET indicates that changes are occurring within the ET structure. Cadaver studies provided the first evidence as to what these changes may be: McCoul et al. endoscopically examined the ET after dilatation, and found linear fissuring with or without submucosal disruption in nearly 60% of ears [7]. Poe et al. found partial-thickness mucosal tears in some BET-treated ETs, but a full-thickness tear in only one [6]. In contrast, Ockermann et al. observed ‘microtears’ within the cartilage, while the mucosa and Ostmann’s fat pad were undamaged [5]. In common with these findings, we identified both mucosal tears and cracks in the apex region of the ET cartilage following BET. The absence of trauma in control specimens, and the blue stain within the mucosal and cartilage tears, suggested that the histological findings were genuine, having occurred during BET.

A single study has reported in vivo histological findings relating to BET: Kivekas et al. found diffuse crush injury and shearing of the mucosa and submucosa following BET, and in three biopsies taken 5 to 12 weeks postoperatively, restoration of largely healthy ciliated pseudo-columnar epithelium was found. It was hypothesised that BET removes irreversibly injured or inflamed cell lining, promoting recovery with normal tissue. This correlates with endoscopic findings in patients where visually scored mucosal inflammation at the nasopharyngeal orifice appears to improve after BET [11, 12]. In our study, it is likely that crushing of the mucosa and submucosa occurred, but it was felt that this could not reliably be assessed in the ET specimens, mainly due to the potential effects of manipulation of the tissue during harvest.

### How does the choice of BET balloon device or technique affect deformation of the ET?

This study demonstrates that balloon size, inflation pressure and balloon insertion depth all impact on the changes induced in the ET during BET, and these are therefore likely to be important variables that determine the clinical effectiveness of the intervention. These technical details have typically been overlooked when comparing or combining BET clinical data, but they may underlie some of the variation seen in outcomes [2], and they will be important considerations during future development of the technique.

### Balloon size

Figure 4 demonstrates a significantly greater dissipation of energy during plastic deformation with the 6.0/6.5-mm balloons, a trend also seen in Fig. 6, suggesting that more plastic deformation occurred with the wider device. Given the findings, with the wider balloons the same tissue must be displaced or crushed more, and/or more tissue must be involved, possibly due to involvement of deeper structures such as cartilage or muscle.

### Depth of insertion

**Fig. 8:**
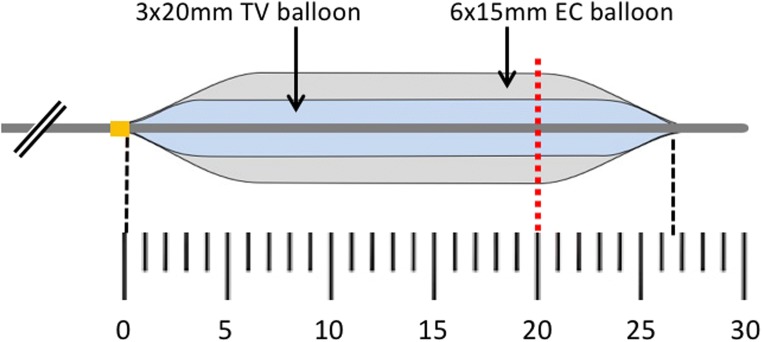
Scale diagram of the 3 × 20 mm TubaVent (TV) balloon overlying the 6 × 15 mm EverCross (EC) balloon (6.5 × 15 mm Sterling Monorail balloon not shown on this figure for clarity). Full insertion of both TubaVent and AERA devices is intended during clinical use. The red dashed line indicates the mean point of the narrowest part of the ET based on histological studies

The cartilaginous ET targeted for dilation with BET is conical in shape, narrowing from a relatively wide nasopharyngeal ostium to the narrowest part of the ET close to the bony-cartilaginous junction. It is perhaps not surprising therefore that plastic deformation of the ET appears to increase with greater depth from the ET nasopharyngeal ostium. Figure 8 shows the balloon dimensions in relation to the narrowest part of the ET, which in adults is located 20.5 ± 4.2 mm from the pharyngeal orifice [13]. It is noticeable that due to the tapering design of the balloon ends (a feature also seen in the AERA balloon not tested), the 20-mm-long balloon does not have an effect on a longer section of the ET than the 15-mm balloon. If the balloons are fully inserted as instructed by the BET device manufactures using either the TubaVent introducer or the AERA insertion marker, during inflation the full diameter of the balloon should sit within or close to the narrowest point of the ET.

Partial insertion of the balloon into the ET will mean that it only sits within the wider, proximal section of the ET, and only the taper, or indeed none of the balloon with reach the narrowest point. As might therefore be predicted, plastic deformation with the smaller, 3.0-mm diameter balloon was very limited when it was inserted only to 10 mm, and progressively increased with insertion to 15 and then 20 mm. This suggests that care must be taken to fully insert BET devices during clinical use, and in cases where preoperative CT has been performed, this may also provide a guide to insertion depth.

Given reports of improvements in OETD following laser treatment of the nasopharyngeal end of the ET, it may be that BET outcomes could be improved if the device had an effect on the whole length of the ET. It is also possible that laser treatment could be complementary to BET, targeting different areas of the tube.

### Inflation pressure

The majority of the plastic deformation occurred at relatively low inflation pressures, at or below 5 bar, and while deformation appeared to continue up to 10 bar with all balloons tested, this was less marked. BET and angioplasty balloons are designed to be non-compliant, meaning a designated size is reached at relatively low pressure, with minimal size increase with additional pressure. Our measurements confirmed this to be the case with the devices used in this study, and this may explain why higher pressures cause limited additional plastic deformation. In the absence of reported significant pressure-related complications, and with some ongoing changes up to 10 bar, currently this appears a reasonable target inflation pressure. However, BET can be performed under local anaesthesia [14], and lower-pressure inflation could be considered in these cases to reduce patient discomfort.

### Number of inflations

Plastic deformation was only seen on the first inflation, with no further changes occurring during the second or third inflation. This suggests that one inflation is sufficient, and the multiple inflations proposed by some clinicians are unlikely to add clinical benefit, and therefore may unnecessarily prolong the procedure.

### How might BET-induced changes improve ET function in cases of OETD?

Although clinical studies of BET have been of limited size and quality, BET appears to provide at least a short-term symptomatic improvement in patients with OETD [2]. Clinical data have also shown increased passive ET opening with Valsalva [12, 15, 16], a marked improvement in active ET opening with swallowing [17], and normalisation of tubomanometry *R* values [18, 19]. Based on the experimental findings, the mechanisms for these clinical improvements can be hypothesised.

### Immediate changes—tissue trauma

If mucosa, cartilage or even muscle is crushed, the ET lumen may become more open at rest, or open wider during paratubal muscle contraction. According to Poiseuille’s law, the wider lumen would reduce the resistance to air flow, possibly improving ET ventilatory function.

An alternative, or possibly complementary, explanation for the experimental findings is that BET alters the stiffness (compliance) of part or all of the ET, through the crushing of soft tissues, or the mucosal tears and cartilage cracks as seen histologically. Of note, the cracks occurred in a region of the ET rich in elastin fibres that is thought to act as a hinge during opening [20]. A number of studies have suggested that ET compliance is an important determinant for its function, with low compliance (high stiffness) associated with OETD and middle ear effusion in adults [21–23]. Modelling also suggests that an increase in ET compliance may enhance active ET opening, with mucosal stiffness of greater influence than muscle function or cartilage stiffness [24, 25]. The relationship between compliance and ET function may be more complex in children, however [21, 26, 27].

The findings from this study could be consistent with an increase in lumen diameter following BET, or with an increase in ET compliance. The relative contribution of these acute structural and mechanical changes to ET function following BET in vivo remains unclear, and post-operative swelling is also likely to occur soon after BET possibly affecting ET mechanical properties.

### Delayed changes—regeneration

A small number of studies have found that the improvement in ET function after BET is not immediate, but emerges after a period of 6 to 8 weeks [18, 28]. This timescale suggests that a process of healing or scarring is occurring to improve the function*.*

Looking to analogues of BET in other tissues, Modi et al. performed an in vivo assessment of balloon dilation of the subglottic airway in a rabbit model [29], and while differences in structure exist, both the subglottis and ET have a cartilaginous skeleton lined by respiratory epithelium and mucosa. Thirty days following dilation, they found regeneration of a normal epithelial lining, with submucosal fibrosis present in many specimens, particularly where larger balloon sizes had been used. It may be that submucosal fibrosis occurs following BET, possibly stiffening the ET.

In cases of otitis media, the ET mucosa is thickened with numerous glands, and the underlying submucosa contains large numbers of macrophages, plasma cells and lymphocytes [30]. The crushing of this tissue and restoration of healthy mucosa as described by Kivekas et al. has a number of possible effects. Healthy mucosa will be thinner, facilitating ET opening, and the normal function of cilia and mucus production may aid the clearance of secretions from the middle ear [31]. In addition, ET mucosa generates a surfactant that reduces surface tension and acts as an anti-adhesive, reducing the ET opening pressure [32]. There is evidence that ET surfactant may be reduced in OME [32, 33], and the restored healthy mucosa may secrete more surfactant, thus improving ET opening.

ET cartilage displays features of both fibrous and elastic tissue types [20], but the hinge portion in which the cracks were found following BET is most similar to the elastic cartilage of the pinna and epiglottis. It is likely that there will be some healing of these cracks, with comparable elastic cartilage elsewhere tending to repair slowly with a dominance of type 1 collagen, and loss of normal tissue architecture that may affect ET compliance [34].

### Limitations and future possibilities

This work represents the largest study of BET in cadavers, though sample sizes were necessarily limited. A striking feature of the findings was the consistency between different specimens, with every ear demonstrating some degree of plastic and elastic deformation, and a statistically significant difference in energy dissipation found between balloon sizes. However, there were an inadequate number of specimens to enable a link to be established between histologically identified trauma and the results of pressure-volume loops. All cadaver specimens used were adults, typically of older age, and as changes may occur in Eustachian tube cartilage with ageing (such as focal calcification) it is possible that results could differ in young adults.

The main limitation of this cadaver-based work was the inability to study the effect of tissue healing and remodelling following BET, or the clinical impact of the mechanical changes. Following further development, the high-pressure syringe driver could be used during BET in vivo to allow quantification of the energy dissipation, and therefore an assessment of the plastic deformation occurring during the first inflation. These results could then be correlated with clinical outcome measures at follow-up intervals, to determine if the extent of deformation measured has a clinical correlate. If the two were found to be related, deformation could be maximised through experimental modification of the device (size, shape, materials) or technique (insertion pressure and duration of inflation) in cadavers. It should be noted that in theory if balloon dilation was performed excessively, a permanently open or patulous Eustachian tube may result, which is a condition equally as problematic as OETD. While this has not been reported clinically using the balloon sizes or techniques tested in this study, it is another area that requires clinical correlation when designing future devices and techniques.

## Conclusions

This study demonstrates the effective application of several engineering methods to a clinical problem, where mechanical data have provided an insight into the mechanism of a surgical intervention that can now be used to direct clinical assessment and future development.

The precise pressure-volume assessment and data analysis employed in this study have not been previously described, but appear to allow quantification of tissue deformation that may be of relevance in the assessment many applications of balloon dilation, such as for vascular or tracheal stenosis. These novel methods were paired with model material tests that helped to validate the correlation of our cadaveric experimental findings with different aspects of tissue deformation occurring in the ET.

We have now been able to demonstrate that BET causes deformation of the ET and a change in its mechanical properties, likely in part due to cracking of the cartilaginous ET skeleton and tearing of its mucosal lining. The extent of deformation occurring differs significantly between the two commercially available devices for which we have published data, with more deformation seen with the larger, 6.0/6.5-mm-diameter balloon. Full balloon insertion is important if maximum deformation is to be achieved, whereas most deformation occurs at pressure well below those used clinically, and only a single inflation appears to be beneficial. Until clinical efficacy is demonstrated, the BET technique should be standardised as much as possible when designing trials, to reduce the number of variables between studies, facilitating meaningful comparison of trial outcomes and meta-analysis of data.

The link between our biomechanical and histological findings and the clinical outcome remains a hypothesis, and importantly we currently we do not know if the increased deformation seen with larger balloons and higher pressures is of clinical benefit. However, we have now established a mechanical method to quantify ET deformation during BET, aiding our understanding of how future clinical studies should be designed and interpreted.

## Electronic supplementary material

Supplementary figure A. Method of calculation for the energy dissipation during plastic deformation of the ET. The balloon-only data are shown, but not used in the calculation. (PNG 491 kb)

High resolution image (TIFF 3716 kb)

Supplementary figure B. Measured and manufacturer stated balloon dimensions. TV = TubaVent, EC = EverCross, SM = Sterling Monorail (PNG 286 kb)

High resolution image (TIFF 3431 kb)

## References

[1] Browning GG, Gatehouse S (1992). The prevalence of middle ear disease in the adult British population. Clin Otolaryngol Allied Sci.

[2] Huisman JML, Verdam FJ, Stegeman I, de Ru JA (2017) Treatment of Eustachian tube dysfunction with balloon dilation: a systematic review. Laryngoscope 10.1002/lary.26800 28799657

[3] Norman G, Llewellyn A, Harden M, Coatesworth A, Kimberling D, Schilder A, McDaid C (2014). Systematic review of the limited evidence base for treatments of Eustachian tube dysfunction: a health technology assessment. Clin Otolaryngol.

[4] Fowkes FG, Gillespie IN (2000) Angioplasty (versus non surgical management) for intermittent claudication. Cochrane database of systematic reviews (Online):CD000017 10.1002/14651858.CD000017 10796469

[5] Ockermann T, Reineke U, Upile T, Ebmeyer J, Sudhoff HH (2010) Balloon dilation eustachian tuboplasty: a feasibility study. Otol Neurotol 31:1100–1103 10.1097/MAO.0b013e3181e8cc6d 20657335

[6] Poe DS, Hanna BM (2011). Balloon dilation of the cartilaginous portion of the eustachian tube: initial safety and feasibility analysis in a cadaver model. Am J Otolaryngol.

[7] McCoul ED, Singh A, Anand VK, Tabaee A (2012). Balloon dilation of the eustachian tube in a cadaver model: technical considerations, learning curve, and potential barriers. Laryngoscope.

[8] Kivekas I, Chao WC, Faquin W, Hollowell M, Silvola J, Rasooly T (2015). Histopathology of balloon-dilation Eustachian tuboplasty. Laryngoscope.

[9] Bluestone CD (2005). Eustachian tube structure, function, role in otitis media.

[10] Jufas N, Treble A, Newey A, Patel N (2016). Endoscopically guided transtympanic balloon catheter dilatation of the Eustachian tube: a cadaveric pilot study. Otol Neurotol.

[11] Poe DS, Pyykko I (2011). Measurements of Eustachian tube dilation by video endoscopy. Otol Neurotol..

[12] Silvola J, Kivekäs I, Poe DS (2014). Balloon dilation of the cartilaginous portion of the Eustachian tube. Otolaryngol Head Neck Surg.

[13] Sudo M, Sando I, Ikui A, Suzuki C (1997). Narrowest (isthmus) portion of Eustachian tube: a computer-aided three-dimensional reconstruction and measurement study. The Annals of otology, rhinology, and laryngology.

[14] Luukkainen V, Kivekas I, Hammaren-Malmi S, Rautiainen M, Poyhonen L, Aarnisalo AA (2017). Balloon Eustachian tuboplasty under local anesthesia: is it feasible?. Laryngoscope.

[15] Xiong H, Liang M, Zhang Z, Xu Y, Ou Y, Chen S (2016). Efficacy of balloon dilation in the treatment of symptomatic Eustachian tube dysfunction: one year follow-up study. Am J Otolaryngol.

[16] Jurkiewicz D, Bien D, Szczygielski K, Kantor I (2013). Clinical evaluation of balloon dilation Eustachian tuboplasty in the Eustachian tube dysfunction. Eur Arch Otorhinolaryngol.

[17] Wanscher JH, Svane-Knudsen V (2014). Promising results after balloon dilatation of the Eustachian tube for obstructive dysfunction. Dan Med J.

[18] Schroder S, Lehmann M, Ebmeyer J, Upile T, Sudhoff H (2015). Balloon Eustachian Tuboplasty (BET): our experience of 622 cases. Clin Otolaryngol.

[19] Gurtler N, Husner A, Flurin H (2014) Balloon dilation of the Eustachian tube: early outcome analysis. Otol Neurotol 10.1097/MAO.0000000000000631 25356762

[20] Matsune S, Sando I, Takahashi H (1992). Elastin at the hinge portion of the eustachian tube cartilage in specimens from normal subjects and those with cleft palate. The Annals of otology, rhinology, and laryngology..

[21] Takahashi H, Hayashi M, Honjo I (1987). Compliance of the eustachian tube in patients with otitis media with effusion. Am J Otolaryngol.

[22] Kaneko A, Doi T, Hosoda Y, Iwano T, Yamashita T (1996). Direct measurement of Eustachian tube compliance. Acta Otolaryngol.

[23] Kaneko A, Hosoda Y, Doi T, Tada N, Iwano T, Yamashita T (2001). Tubal compliance--changes with age and in tubal malfunction. Auris Nasus Larynx.

[24] Sheer FJ, Swarts JD, Ghadiali SN (2012). Three-dimensional finite element analysis of Eustachian tube function under normal and pathological conditions. Med Eng Phys.

[25] Sheer FJ, Swarts JD, Ghadiali SN (2010). Finite element analysis of Eustachian tube function in cleft palate infants based on histological reconstructions. The Cleft palate-craniofacial journal : official publication of the American Cleft Palate-Craniofacial Association.

[26] Takahashi H, Honjo I, Fujita A (1994). Eustachian tube compliance in cleft palate--a preliminary study. Laryngoscope.

[27] Miura M, Takahashi H, Honjo I, Hasebe S, Tanabe M (1997). Influence of the upper respiratory tract infection on tubal compliance in children with otitis media with effusion. Acta Otolaryngol.

[28] Leichtle A, Hollfelder D, Wollenberg B, Bruchhage KL (2017) Balloon Eustachian tuboplasty in children. European archives of oto-rhino-laryngology : official journal of the European Federation of Oto-Rhino-Laryngological Societies 10.1007/s00405-017-4517-8 28283791

[29] Modi VK, Visaya JM, Ward RF (2015) Histopathological effect of balloon dilation in a live rabbit: implications for the pediatric airway. Laryngoscope 125(Suppl 6):S1–S11 10.1002/lary.25425 26153243

[30] Lim DJ (1979). Normal and pathological mucosa of the middle ear and eustachian tube. Clinical otolaryngology and allied sciences.

[31] Bluestone CD (2005). Eustachian tube structure, function, role in otitis media.

[32] McGuire JF (2002). Surfactant in the middle ear and Eustachian tube: a review. Int J Pediatr Otorhinolaryngol.

[33] Zhu ZH, Shan YJ, Han Y, Zhu LW, Ma ZX (2013). Pathological study of otitis media with effusion after treatment with intranasal pulmonary surfactant. Laryngoscope.

[34] Zhu X, Tang Y, Chen J, Xiong S, Zhuo S, Chen J (2013). Monitoring wound healing of elastic cartilage using multiphoton microscopy. Osteoarthr Cartil.

