# Supplementary figures and images for: The mechanism of balloon Eustachian tuboplasty: a biomechanical study
Source: Med Biol Eng Comput. 2020 Jan 17;58(4):689–99. doi: 10.1007/s11517-020-02121-z (PMC7156363; doi:10.1007/s11517-020-02121-z)

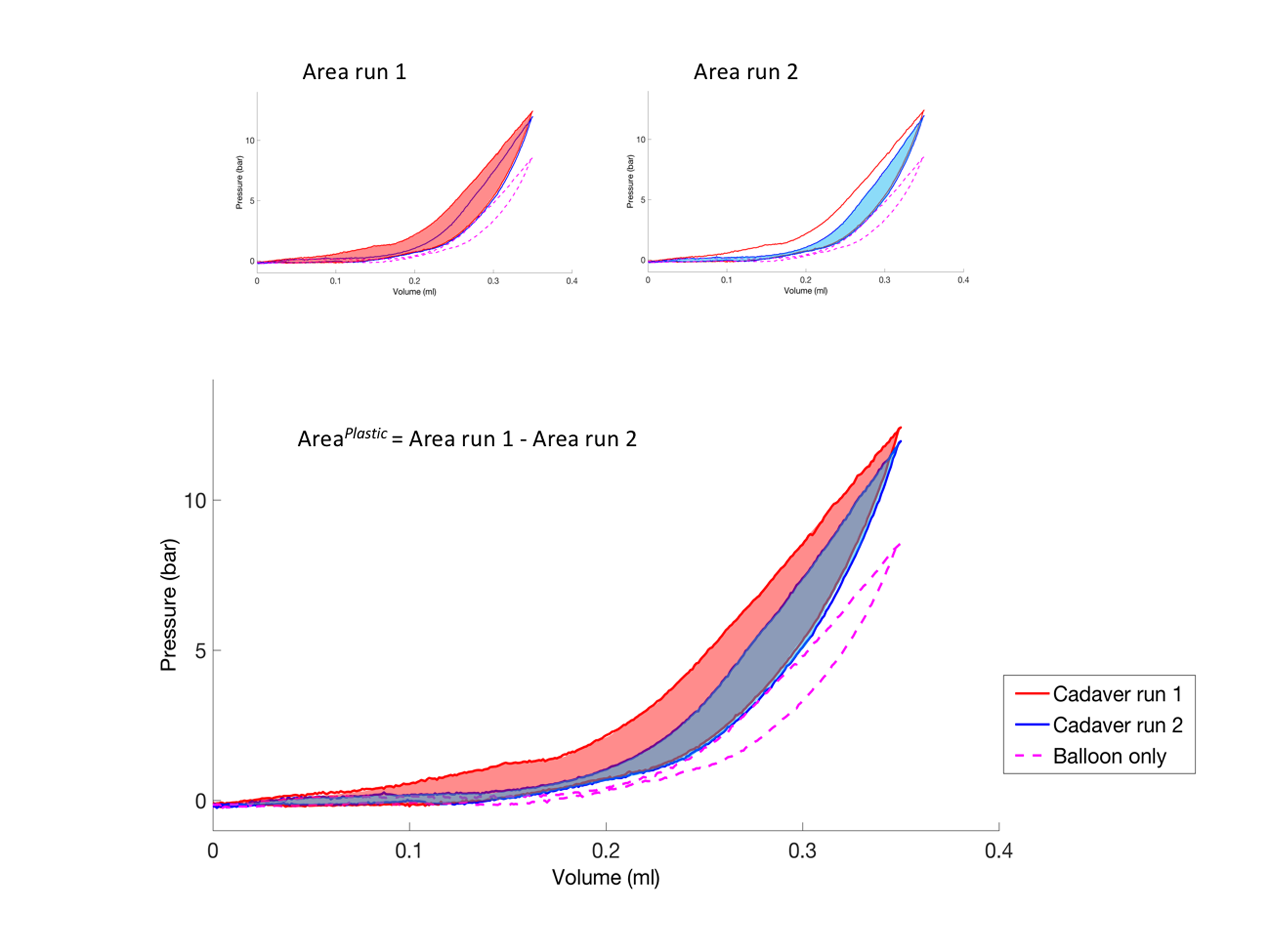

Supplement: Supplementary file 1 — Method of calculation for the energy dissipation during plastic deformation of the ET. The balloon-only data are shown, but not used in the calculation. (PNG 491 kb) [file 11517_2020_2121_Fig9_ESM.png]

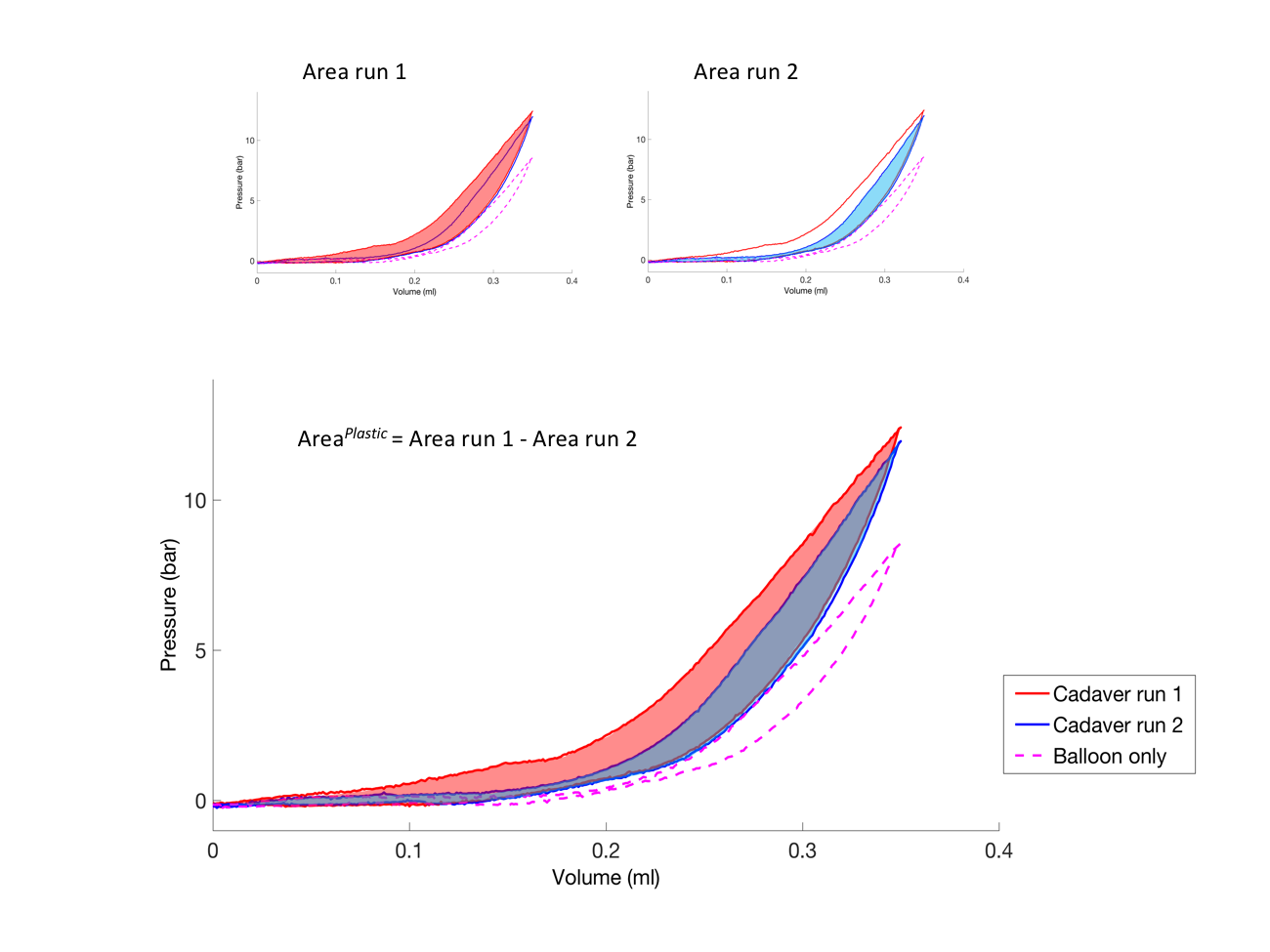

Supplement: Supplementary file 2 — High resolution image (TIFF 3716 kb) [file 11517_2020_2121_MOESM1_ESM.tiff]

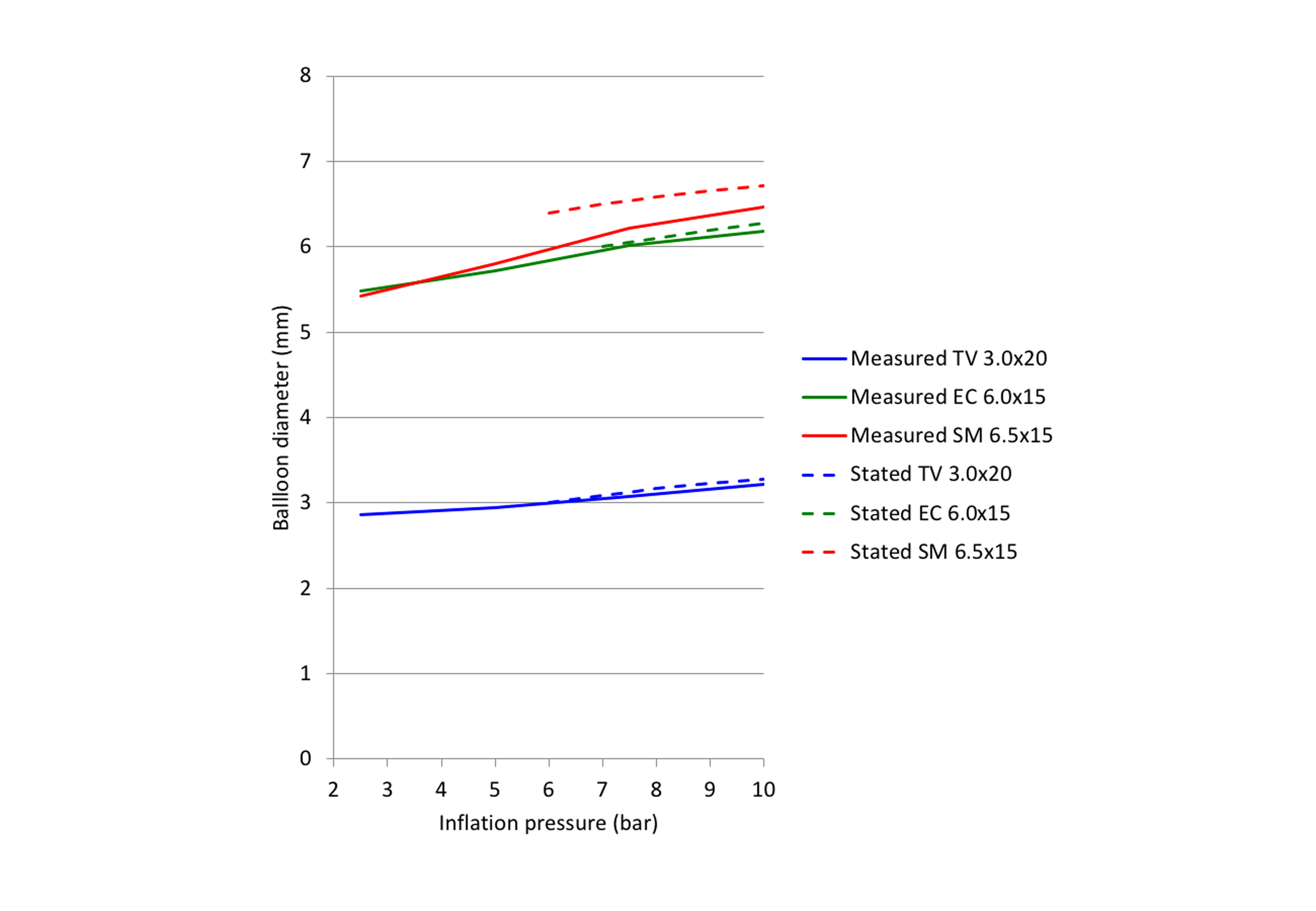

Supplement: Supplementary file 3 — Measured and manufacturer stated balloon dimensions. TV = TubaVent, EC = EverCross, SM = Sterling Monorail (PNG 286 kb) [file 11517_2020_2121_Fig10_ESM.png]

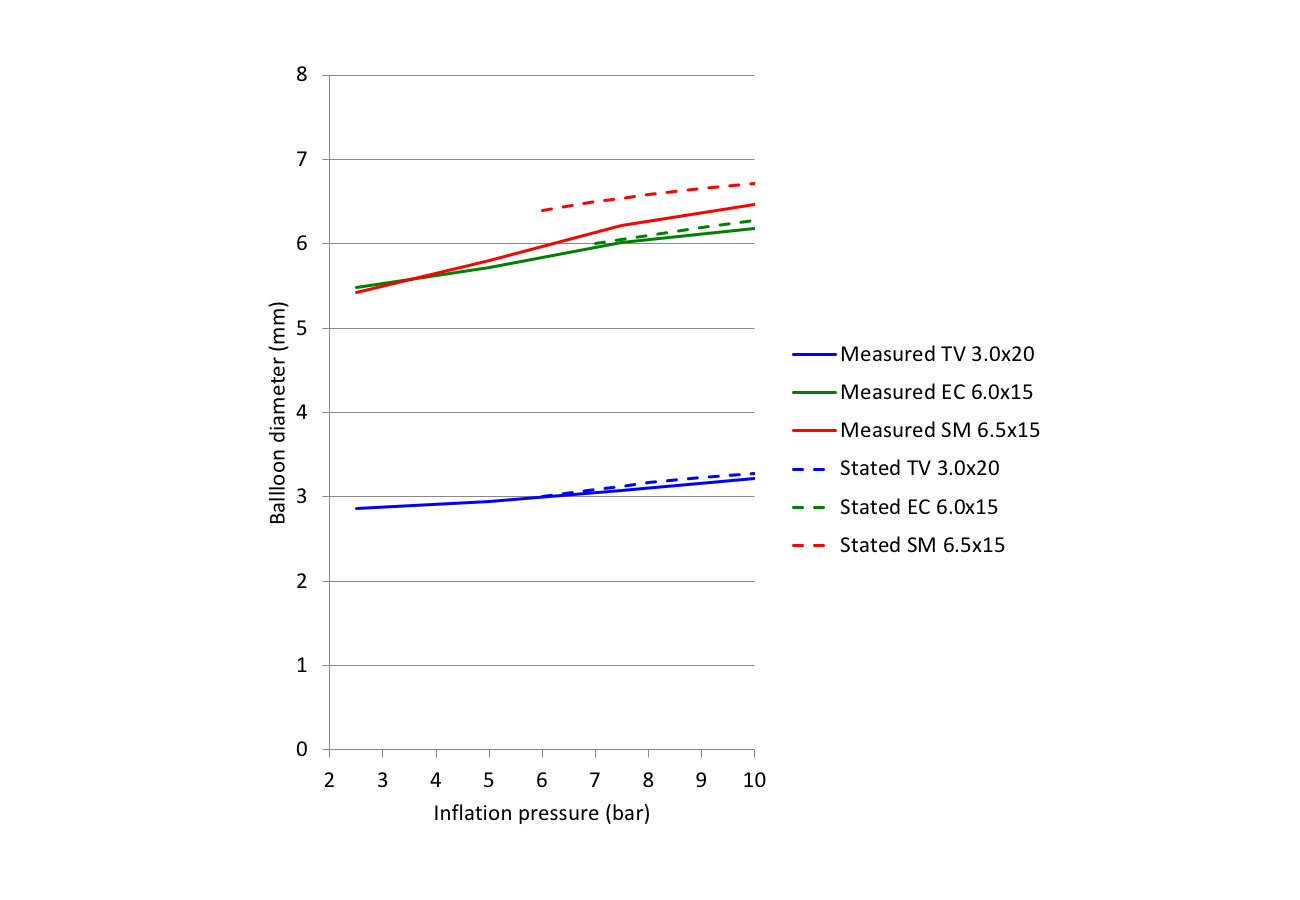

Supplement: Supplementary file 4 — High resolution image (TIFF 3431 kb) [file 11517_2020_2121_MOESM2_ESM.tiff]
